# Continuous infusion of antibiotics in the critically ill: The new holy grail for beta-lactams and vancomycin?

**DOI:** 10.1186/2110-5820-2-22

**Published:** 2012-07-02

**Authors:** Bruno Van Herendael, Axel Jeurissen, Paul M Tulkens, Erika Vlieghe, Walter Verbrugghe, Philippe G Jorens, Margareta Ieven

**Affiliations:** 1 Department of Microbiology, Antwerp University Hospital, Edegem, Belgium; 2 Department of Microbiology, GZA St. Vincentius Hospital, Antwerp, Belgium; 3 Cellular and Molecular Pharmacology and Centre for Clinical Pharmacy, Louvain Drug Research Institute, Université catholique de Louvain, Brussels, Belgium; 4 Department of Tropical Diseases, Antwerp University Hospital, Edegem, Belgium; 5 Institute of Tropical Medicine, Antwerp, Belgium; 6 Department of Intensive Care Medicine, Antwerp University Hospital, Edegem, Belgium

**Keywords:** Continuous infusion, Intermittent infusion, Vancomycin, Beta-lactam, Antibiotic(s), Critically ill, Pharmacokinetic/pharmacodynamic

## Abstract

The alarming global rise of antimicrobial resistance combined with the lack of new antimicrobial agents has led to a renewed interest in optimization of our current antibiotics. Continuous infusion (CI) of time-dependent antibiotics has certain theoretical advantages toward efficacy based on pharmacokinetic/pharmacodynamic principles. We reviewed the available clinical studies concerning continuous infusion of beta-lactam antibiotics and vancomycin in critically ill patients. We conclude that CI of beta-lactam antibiotics is not necessarily more advantageous for all patients. Continuous infusion is only likely to have clinical benefits in subpopulations of patients where intermittent infusion is unable to achieve an adequate time above the minimal inhibitory concentration (T > MIC). For example, in patients with infections caused by organisms with elevated MICs, patients with altered pharmacokinetics (such as the critically ill) and possibly also immunocompromised patients. For vancomycin CI can be chosen, not always for better clinical efficacy, but because it is practical, cheaper, associated with less AUC_24h_ (area under the curve >24 h)-variability, and easier to monitor.

## Background

Antimicrobial resistance is emerging worldwide [1]. In addition there is a dramatic lack of new antimicrobial agents being explored in phase 2 or 3 clinical trials, especially for Gram-negative organisms, and development of an antimicrobial with a genuinely novel mechanism of action is estimated to take years [2].

These evolutions have spurred interest in maximizing the effectiveness of our current antimicrobial armamentarium to retain its activity for the years to come. One of the ways to achieve this is through optimization of antibiotic dosing regimens based on pharmacokinetic/pharmacodynamic (PK/PD) principles. Animal studies and PK/PD studies suggest that efficacy of beta-lactam antibiotics is better when administered as continuous infusion (CI) or prolonged infusion than when administered as intermittent infusion (II), yet this remains to be proven in clinical outcome studies. CI of vancomycin also is increasingly popular, albeit more for practical reasons and aiming to limit toxicity rather than for reasons related to PK/PD and expected clinical efficacy. In this paper, we therefore focus on the clinical evidence for CI of both the beta-lactam antibiotics and vancomycin in critically ill patients.

## Review

### β-lactam antibiotics

Beta-lactam antibiotics are “time-dependent antibiotics”. Their maximal killing rate is achieved at concentrations that are only about four times the minimal inhibitory concentration (MIC), which is a relatively low concentration if considering the actual serum levels that are achieved by intermittent administration (typical C_max_ will reach values > 80 mg/L), and the current clinical breakpoints of beta-lactams (i.e., the highest MIC that an antibiotic may show against a given organism while keeping a high likelihood of clinical success) that rarely exceed 16 mg/L. Concentrations higher than four times the MIC do not lead to more bacterial killing [3-5]. For beta-lactam antibiotics, therefore, not the peak concentration (nor the area under the curve as such) correlates best with efficacy, but rather the fraction of the time during which the serum concentration of the antibiotic exceeds the MIC between two successive administrations (T > MIC) [3,6]. For most non-life-threatening infections in nonimmunocompromised patients, a T > MIC of 40–50% is sufficient for successful clinical outcome. Animal studies with *Streptococcus pneumoniae* infected nonneutropenic mice showed that survival reached a plateau (with excellent survival) if serum levels exceeded the MIC for no longer than 40–50% of the dosing interval (reviewed in [7]). A recent human study, however, suggests that for critically ill patients clinical outcome might be better if T > MIC approximates the full 100% [8]. Although T > MIC is the most important parameter it also is important to achieve a steady state concentration (Css) of at least 2.5 times the MIC and preferably 4 times the MIC. Mouton and Hollander [9] showed that a Css equal to or slightly above the MIC during continuous infusion can lead to selection of resistant subpopulations *in vitro*. In a study from Alou and coworkers [10], the authors advise a Css/MIC ratio of at least 2.5, because they found bacterial regrowth when the Css/MIC ratio was 1.26, but not when it was 2.5.

Several PK/PD studies with different beta-lactam antibiotics have shown that their administration by CI significantly increases the likelihood of maintaining serum levels above the MIC compared with II, most notably when considering the highest clinical susceptibility breakpoints [10-18]. This is well illustrated in study by Alou and coworkers [10] who used a pharmacokinetic computerized model to simulate concentrations of ceftazidime in human serum after administration of 6 g/day using either CI or II. Using isolates with MICs of 8, 16, and 32 mg/L, the authors found a T > MIC of 99.8%, 69%, and 47.6%, respectively, for the bolus regimen and 100% for all strains with CI. Therefore, a first population at risk to consider for CI is patients with infections caused by pathogens with MICs close to the susceptibility breakpoint. A second at-risk population are critically ill patients where altered physiology with increased volume of distribution (V_d_) and increased drug clearance can lead to lower initial as well as faster decreasing serum concentrations than expected for typical drug dosages [19,20]. This adds a direct pharmacokinetic argument to the well-accepted necessity to ensure a longer T > MIC in critically ill patients compared with other patients for pharmacodynamic reasons [8]. A third population at risk might be neutropenic patients. A series of animal studies [21-24] comparing clinical outcome after CI versus II in rats with pulmonary infection shows a clear benefit in neutropenic rats in favor of CI. The daily dose needed to protect 50% of the rats from mortality was 15 times lower with CI. This effect disappeared in nonneutropenic animals.

Patients in the ICU are at risk for infections with higher MICs. For these infections, it can be quite difficult to achieve acceptable PK/PD targets with classical intermittent dosing regimens. Even when using CI, the combination of pathogens with MICs close to the susceptibility breakpoint and critically ill patients with lower than expected Css (because of increased Vd and increased drug clearance) can lead to critically low Css/MIC ratios below the advised ratio of 2.5 to 4. An overview of studies [11,15,25-33] investigating PK-parameters of continuous infusion of different antimicrobials in critically ill patients is shown in Table 1. In most of these studies, the Css/MIC ratio is well above 2.5. However, for cefepime (at a submaximal dosage of 4 g/d) [31] and piperacillin (at a submaximal dosage of 12 g/d) [33], the ratio became problematically low for pathogens with MICs close to the susceptibility breakpoint with subsequent risk of therapeutic failure or emergence of resistance. Yet, for any MIC, continuous infusion has a higher likelihood of attaining PK/PD targets than II. So when starting empirical therapy, CI is the safer choice. If however after culture the pathogen proves to be resistant (or even in the intermediate range) another antimicrobial should be chosen. Obtaining a MIC for the causative pathogen in case of severe infection will give even more information than relying on “susceptible/intermediate/resistant” categories only. The value of the MIC can be directly compared with the breakpoints and can be used to guide therapy.

**Table 1:** Overview of studies investigating PK-parameters of continuous infusion of different antimicrobials in critically ill patients

**Antibiotic**	**Mean Css (mg/L)**	**Enterobacteriaceae**				***Pseudomonas aeruginosa***	
		**EUCAST**		**CLSI**		**EUCAST and CLSI**	
		**Breakpoint**	**Ratio: Css/highest possible MIC within susceptibility range**	**Breakpoint**	**Ratio: Css/highest possible MIC within susceptibility range**	**Breakpoint**	**Ratio: Css/highest possible MIC within susceptibility range**
Ceftazidime [25]6 g/24 h	75	≤1	75	≤4	18.75	≤8	9.37
Ceftazidime [26]4.5 g/24 h	47	≤1	47	≤4	11.75	≤8	5.87
Ceftazidime [27]3 g/24 h	30	≤1	30	≤4	7.5	≤8	3.75
Ceftazidime [28]60 mg/kg/d	19	≤1	19	≤4	4.75	≤8	2.37
Ceftazidime [29]4 g/24 h	40	≤1	40	≤4	10	≤8	5
Ceftazidime [11]6 g/24 h	63	≤1	63	≤4	15.75	≤8	7.87
Cefepime [30]4 g/24 h	41	≤1	41	≤8	5.13	≤8	5.13
Cefepime [31]4 g/24 h	13.5	≤1	13.5	≤8	1.68	≤8	1.68
Piperacillin [32]13.5 g/24 h	35	≤8	4.37	≤16	2.18	≤16	2.18
Piperacillin [33]12 g/24 h	18	≤8	2.25	≤16	1.125	≤16	1.125
Meropenem [15]3 g/24 h	7	≤2	3.5	≤1	7	≤2	3.5

EUCAST = European Committee on Antimicrobial Susceptibility Testing; CLSI = Clinical and Laboratory Standards Institute; Css = steady state concentration; MIC = minimal inhibitory concentration.

### General clinical value of CI of β-lactam antibiotics

In 2009, a meta-analysis was published of all randomized controlled trials (RCT) from 1950 through November 2007 comparing the clinical benefits of CI regimens of beta-lactam antibiotics with II regimens [34]. The meta-analysis included 14 RCTs with a total of 846 patients [16,17,25,35-44]. No difference was found for mortality or clinical cure between II or CI. Yet, all but one of the included studies used a higher drug dose in the bolus group than in the CI group, and the authors concluded that CI of a lower dosed beta-lactam antibiotic might lead to the same clinical results as II of the same higher dosed antibiotic. Apart from the different daily dosages, another important limitation was that most studies analyzed a heterogeneous population with underrepresentation of the critically ill, a population where CI is expected to be advantageous.

### Penicillins

For penicillin G and flucloxacillin, most data come from observational, noncontrolled studies often in home-based care settings [45-47]. Both antibiotics proved to be safe when given in CI and achieved an excellent clinical cure rate in stable, noncritically ill patients. Only one study included critically ill patients, but CI was only started after clinical improvement was achieved with II [47]. All previous studies had no comparator group of patients with II. One retrospective study did compare outcome between CI (n = 78) and II (n = 28) of oxacillin for treatment of MSSA (methicillin susceptible *Staphylococcus aureus*) endocarditis. They found similar results for mortality (8% vs. 10%, *p* = 0.7) and length of stay, but microbiological cure at 30 days, defined as “no positive cultures within 30 days of the end of treatment”, differed significantly with odds ratio of 3.8 in favor of CI [48].

Five clinical trials have been published in regards to CI of piperacillin-tazobactam (Table 2). Two of these found no difference for clinical cure rates between CI and II of piperacillin-tazobactam [42,49], although Grant et al. [49] concluded that the costs in the CI group were significantly lower than in the II group. These two studies did not include critically ill patients. Others observed that the severity of illness, measured by APACHE II scores, decreased more rapidly in the CI group of septic patients even though this group received a lower daily dosage [16]. Two retrospective cohort studies described higher rates of clinical cure in the CI arm, but only in subpopulations with the most critically ill patients [50] or with infections caused by more resistant organisms [51]. The last three studies were performed with critically ill, intensive care unit (ICU) patients.

**Table 2:** Characteristics of studies comparing outcome for continuous versus intermittent administration of piperacillin

**Study**	**Drug**	**Patient population**	**Dosage**	**Study type**	**Outcome measure**	**Outcome**	**Remarks**
**Grant 2002**[49]	Piperacillin-tazobactam	Hospitalized patients	12 g/d CI (n = 47) vs. 4 x 3 g/d II (n = 51)	Prospective, open- label controlled study	Clinical cure Microbiological cure Days to defervescence Level 1 costs Level 2 costs	NS NS CI < II (*p * = 0.012) NS CI < II (*p * = 0.028)	
**Lau 2006**[42]	Piperacillin-tazobactam	Complicated intra- abdominal infections	12 g/d CI (n = 130) vs. 4 x 3 g/d II (n = 132)	RCT	Clinical cure Mortality Adverse events	NS NS NS	
*Rafati 2006[16]	Piperacillin	Septic, critically ill patients	8 g/d CI (n = 20) vs. 4 x 3 g/d II (n = 20)	RCT	Mortality Rate of decrease APACHE II score Days to defervescence	NS CI > II NS	Lower antimicrobial dose in CI group
*Lodise 2007[50]	Piperacillin- tazobactam	Pseudomonas aeruginosa infections, including both ICU patients (n = 126) and non-ICU patients (n = 68)	3 x 3.375 g/d in extended infusions of 4 h (n = 102) vs. 4 or 6 x 3.375 g/d II (n = 92)	Retrospective cohort study	14-day mortality APACHE II < 17 APACHE II ≥ 17 Length of stay: APACHE II < 17 APACHE II ≥ 17	NSExtended infusion < II (p = 0,04) NSExtended infusion < II (p = 0,02)	Lower antimicrobial dose in extended infusion group Significant outcome differences only in subpopulation with high severity of illness (APACHE II ≥ 17)
*Lorente 2009[51]	Piperacillin- tazobactam (+ tobramycin 7 mg/kg/d)	VAP	16 g/d CI (n = 37) vs. 4 x 4 g/d II (n = 46)	Retrospective cohort study	Clinical cure: MIC = 4 μg/ml MIC = 8 μg/ml MIC = 16 μg/ml	NSCI > II(*p * = 0.02) CI > II(*p * = 0.02)	Significant outcome differences only in infections caused by pathogens with high MICs

*Studies that included critically ill, ICU patients.

NS = nonsignificant; MIC = minimal inhibitory concentration; CI = continuous infusion; II = intermittent infusion; APACHE II = Acute Physiology and Chronic Health Evaluation; VAP = ventilator-associated pneumonia; RCT = randomized, controlled trial; ICU = intensive care unit.

### Cephalosporins

Studies comparing clinical outcome between CI and II of cephalosporins are listed in Table 3. Four showed comparable clinical cure rates [17,28,38,41]. However, most used a lower daily dose in the CI arm than in the II arm [17,28,38]. In the studies by Hanes and Georges, both CI and II regimens achieved T > MIC > 90%, which explains the comparable outcomes [28,41].

**Table 3:** Characteristics of studies comparing outcome for continuous versus intermittent administration of cephalosporins

**Study**	**Drug**	**Patient population**	**Dosage**	**Study type**	**Outcome measure**	**Outcome**	**Remarks**
**Bodey 1979**[36]	Cefamandole (+ carbenicillin 6 x 5 g/d)	FUO in neutropenic patients	12 g/d CI (n = 74) vs. 4 x 3 g/d II (n = 92)	RCT	Clinical cure	NS	Significant difference in clinical cure (in favor of CI) only in subpopulation with persistent neutropenia (*p * = 0.03)
*Hanes 2000[28]	Ceftazidime	Nosocomial pneumonia in critically ill trauma patients	60 mg/kg/d CI (n = 17) vs. 3 x 2 g/d II (n = 15)	RCT	Length of stay Duration of leucocytosis Days to defervescence	NS NS NS	T > MIC > 90% in both II and CI groupLower antimicrobial dose in CI group
*Nicolau 2001[38]	Ceftazidime (+tobramycin 7 mg/kg/d)	Nosocomial pneumonia in ICU patients	3 g/d CI (n = 17) vs. 3 x 2 g/d II (n = 18)	RCT	Clinical cure Microbiological cure	NS NS	Lower antimicrobial dose in CI group
*Lorente 2007[52]	Ceftazidime (+tobramycin 7 mg/kg)	VAP	4 g/d CI (n = 56) vs. 2 x 2 g/d II (n = 65)	Retrospective, nonrandomized, historical chart review	Clinical cure	CI > II(*p * < 0.001)	
*Roberts 2007[35]	Ceftriaxone	Sepsis	2 g/d CI (n = 29) vs. 1 x 2 g/d II (n = 28)	RCT	Clinical cure- ITT analysis*a priori*	NS CI > II (*p * = 0.04)	Significant difference in clinical cure only in an ‘a priori’ defined subgroup of patients who received at least 4 days of ceftriaxone therapy (to exclude patients that were not ill enough or too ill)
**Van Zanten 2007**[17]	Cefotaxime	COPD exacerbations	2 g/d CI (n = 47) vs. 3 x 1 g/d II (n = 46)	RCT	Clinical cure	NS	Lower antimicrobial dose in CI group
*Georges 2005[41]	Cefepime (+ amikacin 15 mg/kg/d)	Severe pneumonia or bacteremia	4 g/d CI (n = 26) vs. 2 x 2 g/d II (n = 24)	RCT	Clinical cure	NS	

*Studies that included critically ill, ICU patients.

FUO = fever of unknown origin; NS = nonsignificant; RCT = randomized, controlled trial; ITT = intention to treat; CI = continuous infusion; II = intermittent infusion; VAP = ventilator-associated pneumonia; ICU = intensive care unit.

Three studies showed a clinical advantage with continuous infusion; however, mostly only in a subpopulation. Roberts and coworkers [35] compared CI of ceftriaxone versus II in 57 intensive care patients with sepsis and found a significant advantage in favor of CI only in an *a priori*-defined subgroup of patients (n = 50) who received more than 4 days of antibiotic therapy, but not in the intention-to-treat analysis that included all patients (n = 57). This subgroup analysis was performed to exclude patients who were either moribund (too ill) or did not need antimicrobial treatment (not ill enough). A retrospective study by Lorente et al. showed a greater clinical cure rate for CI versus II of ceftazidime (and tobramycin) for the treatment of critically ill patients with ventilator-associated pneumonia (VAP) [52]. Both arms received a low total daily dose of 4 g/d (instead of the standard dosage of 6 g/d). This could have influenced results, because with suboptimal dosages the chances to attain an acceptable T > MIC are much higher with CI. Finally, an older study [36] found that continuous cefamandole infusion (with carbenicillin in II) achieved a greater effectiveness than intermittent carbenicillin infusion (with carbenicillin in II) in a subgroup of patients with persistent severe neutropenia.

### Carbapenems

A retrospective cohort study with 89 patients [53] comparing CI versus II of meropenem for the treatment of VAP due to Gram-negative bacilli found a significantly better clinical cure rate in the group with CI, especially when used for *Pseudomonas* species and other pathogens with elevated MIC values. The authors used 6-h infusions, although it is generally not advised to give meropenem in infusions longer than 3–4 h for reasons of stability. Sakka conducted a study in which 20 critically ill patients with pneumonia were randomized to receive either a normal dose (3 x 1 g/d) of imipenem-cilastatin by II (n = 10) or a lower dose (2 g/d) by CI (n = 10) [44]. Both regimens achieved excellent target attainment with T > MIC 100% for all patients, which explains the similar outcomes with one patient who died in the CI group and two patients in the II group. Both studies are listed in Table 4.

**Table 4:** Characteristics of studies comparing outcome for continuous versus intermittent administration of carbapenems

**Study**	**Drug**	**Patient population**	**Dosage**	**Study type**	**Outcome measure**	**Outcome**	**Remarks**
*Lorente 2006[53]	Meropenem	VAP with gram negative bacilli	4 g/d CI (n = 42) vs. 4 x 1 g/d (n = 47)	Retrospective cohort study	Clinical cure	CI > II(*p * < 0.001)	
*Sakka 2007[44]	Imipenem-cilastatin	Nosocomial pneumonia	2 g/d CI (n = 10) vs. 3 x 1 g/d (n = 10)	RCT	Clinical cure	NS	Lower antimicrobial dose in CI groupT > MIC = 100 % in both II and CI group

*Studies that included critically ill, ICU patients.

VAP = ventilator-associated pneumonia; RCT = randomized, controlled trial.

With regards to the threat of the emerging carbapenemase-producing *Enterobacteriaceae*, it has been suggested in a recent review that carbapenemase-producing *Klebsiella pneumoniae* with a MIC up to 4 mg/L (higher than the EUCAST (European Committee on Antimicrobial Susceptibility Testing) susceptibility breakpoint of 2 mg/L and the CLSI (Clinical and Laboratory Standards Institute) susceptibility breakpoint of 1 mg/L can still be treated with carbapenems if they are given in an extended infusion and higher dosage regimen [54]. This could create a treatment option in situations where alternatives are extremely limited.

### Temocillin

Only one study has been published concerning CI of the niche antibiotic temocillin [13]. Temocillin remains stable and therefore active in CI and is compatible with aminoglycosides (but not with several other antibiotics). Moreover, the standard dose of 4 g/d yields stable serum concentrations >16 mg/L, the breakpoint of temocillin for *Enterobacteriaceae*. Unfortunately, the study was not powered to compare clinical outcome. A study using a 6-g/d dosage in the ICU with a measure of clinical outcome is now near completion.

### CI of beta-lactam antibiotics: Discussion

The available clinical evidence, also in critically ill patients, does not show a conclusive and significant benefit in favor of CI. This might be due to the fact that CI is only likely to have clinical benefits in the subpopulation of patients for whom II is unable to achieve an adequate T > MIC: for example, in patients with infections caused by borderline susceptible organisms, patients with elevated drug clearance or increased V_d_ (such as the critically ill), and possibly also immunocompromised patients. These populations are difficult to include in homogenous cohorts, and the underlying pathologies may obscure the final picture. However, it is fair to say that in the small numbers of clinical studies that did show an advantage in favor of CI, this effect was only present or more pronounced in a subpopulation of the most critically ill or patients with infections caused by pathogens with elevated MICs. An important limitation of most of the older studies is that they are primarily designed to prove that a lower dose of an antibiotic given in CI can be equally effective as a higher dose given in an intermittent bolus regimen and consequently use lower dosages in the CI arm. Finally, some studies use a second antibiotic, which could be responsible for bias.

Future studies should ideally include a homogeneous population and PK/PD analysis (including the MICs of the responsible pathogens and therapeutic drug monitoring) to know which patients attain PK/PD targets and how this is linked with either infusion regimen and/or outcome. However, serum level determinations for beta-lactam antibiotics are not routinely available, and therefore, PK/PD analysis has only recently gained attention in the ICU world.

Apart from clinical efficacy, there are some other advantages in favor of CI. Several studies show that the costs associated with CI are lower than with II [49,55]. There also is some evidence that tissue penetration might be better with CI. Roberts et al. found that concentration at the subcutaneous tissue was higher when meropenem was administered by CI than when it was administered by II in critically ill patients [15]. Another study shows that the penetration of ceftazidime in patients with severe abdominal infections was better with CI than with II [26].

### CI of beta-lactam antibiotics: Practical considerations

The antibiotic dosage should be adjusted to achieve a serum steady state target concentration approximately four times the MIC of the pathogen, and a loading dose should be administered to reach a steady state more rapidly. Several formulas have been proposed for calculation of loading dose and total daily dose [56]:

Total daily dosage (mg) = 24 (h) x total body clearance (L/h) x target Css (mg/L)

Loading dose (mg) = target peak concentration (mg/L) x volume of distribution (l)

AUC_24h_ = Total daily dosage /[{(creatinine clearance x 0.79) + 15.4} x 0.06]

However, the value of these formulas is limited in a critically ill population; they presume knowledge of volume of distribution (V_d_) and total body clearance (TBC), which is problematic in an ICU population. In critically ill patients, the V_d_ is elevated and difficult to predict. Second, the TBC of beta-lactams is not always readily predictable from the value of creatinine clearance (even for antibiotics such as ceftazidime that are essentially eliminated by the kidney), and moreover, in the critically ill there is no readily available method to measure accurately the glomerular filtration rate (GFR) [57]. The risk of underdosing is certainly higher than the risk of possible toxicity in ICU patients. Because no clinical or biological variable can predict beta-lactam concentrations in critically ill patients, we agree with other authors that therapeutic drug monitoring of beta-lactam antibiotics might be necessary in this population [58]. Because beta-lactams are rather safe drugs, a practical approach commonly used is to start with a loading dose equal to the first dose normally administered when using II, followed immediately by CI using the same total daily dose as in II (note that in doing so, one creates essentially a single serum peak equivalent to what is obtained repeatedly with II).

Not all beta-lactam antibiotics are stable enough for a 24-h infusion. Piperacillin [59], temocillin [13], and aztreonam [59,60] are stable at room temperature for at least 24 h. Ceftazidime and cefepime are stable for 24 h at 25°C, but only for 8 h and 13 h, respectively, at 37°C [59,61,62]. This means they can be used as 24-h infusion without problems if temperature does not exceed 25°C, but home administration with portable pumps next to the body or hospital administration in regions or periods of warm climate can pose problems because of higher temperatures. Meropenem, doripenem, and especially imipenem are less stable and should only be given as extended 3- or 4-h infusions [63]. Amoxicillin is stable at room temperature for 8 h [64] and penicillin for 12 h [65]. Certain physical or chemical incompatibilities with other drugs for infusion through the same intravenous line (Y-site infusion) have been described. Most notable is the incompatibility with vancomycin for most beta-lactam antibiotics. An overview of all incompatibilities is listed in Table 5. If concomitant administration of Y-site incompatible drugs is necessary, they should be administrated trough separate intravenous lines.

**Table 5:** List of drugs that are incompatible with different time-dependent antibiotics when given trough the same intravenous line (Y-site incompatibilities)

**Time-dependent antibiotic**	**Incompatible drugs (should not be given trough the same intravenous line)**
**Flucloxacillin**[64]	Clarithromycin, lorazepam, midazolam, vancomycin
**Ceftazidime**[61,64]	Acetylcysteine, nicardipine, midazolam, propofol, and vancomycin
**Cefepime**[62,64]	Erythromycin, propofol, midazolam, phenytoin, piritramide, theophylline, nicardipine, N-acetylcysteine, vancomycin, and a concentrated solution of dobutamine
**Piperacillin-tazobactam**[64]	Acyclovir, amiodarone, amphotericin B cholesteryl sulfate complex, azithromycin, dobutamine, ganciclovir, haloperidol, vancomycin
**Meropenem**[64]	Acyclovir, amphotericin B, diazepam, ondansetron, doxycycline
**Imipenem**[64]	Allopurinol, amiodarone, amphotericin B cholesteryl sulfate complex, azithromycin, fluconazole, lorazepam, midazolam,
**Temocillin**[13]	Other beta-lactam antibiotics, vancomycin, ciprofloxacin, clindamycin, propofol, midazolam, nicardipine, ranitidine, vitamin K
**Vancomycin [unpublished data by Ampe E, article in preparation]**	Flucloxacillin, temocillin, piperacillin-tazobactam, cephalosporins, imipenem, moxifloxacin, propofol, valproate, phenytoin, theophylline, furosemide, methylprednisolone

### Vancomycin

Vancomycin has a different PK/PD pattern. Like beta-lactam antibiotics, vancomycin also exhibits slow and time-dependent killing during in vitro experiments. However, unlike the beta-lactams, vancomycin has moderately long postantibiotic effects (i.e., withdrawal of the antibiotic is not immediately followed by bacterial regrowth). Therefore, it becomes less important for serum concentrations to remain above the MIC all the time [66]. Moreover, vancomycin has a much longer serum half-life than most β-lactams, which makes the drug remain above the MIC for most of the administration interval (especially if considering the recently revised clinical breakpoints of vancomycin that have been decreased to ≤2 mg/L for susceptible and to >2 for resistant to avoid recommending the use of vancomycin against organisms with higher MICs that would only poorly respond to the antibiotic). Careful animal studies where T > MIC could be unambiguously separated from other PK parameters (which is difficult to obtain in human studies) showed that it is actually AUC_24h_/MIC (area under the curve over 24 h) that best predicts clinical efficacy [67-70].

AUC_24h_ is a parameter that is more difficult to grasp than the T > MIC, but it is proportional to the total daily dosage (TDD) and inversely proportional to the creatinine clearance (CrCl) [69]. Consequently the AUC_24h_ will increase if a greater TDD is given, irrespective of continuous or intermittent dosing. An AUC_24h_/MIC > 350 was associated with clinical success and an AUC_24h_/MIC > 400 with faster bacterial eradication in patients receiving vancomycin for *Staphylococcus aureus* pneumonia [69]. In continuous infusion, the AUC_24h_ is simply the product of the actual serum concentration x24. Based on the above observations concerning the required AUC_24h_/MIC ratio, it means that a stable serum level of 14 to 18 times the MIC must be maintained to achieve optimal efficacy.

### Clinical value of CI of vancomycin

A prospective, randomized trial in 114 patients treated for severe staphylococcal infections compared outcome, safety, and cost of CI versus II infusion of vancomycin [71]. Outcome and safety were similar between the two groups, but with CI target concentrations were achieved more quickly, there was less variability of AUC_24h_ values, and costs were lower. Vuagnat et al. conducted a nonrandomized, prospective trial with 44 patients who required high-dose vancomycin for treatment of osteomyelitis [72]. Twenty-one patients received vancomycin through II and 23 through CI. Outcome was equal with both regimens but with CI target concentrations were achieved more quickly, there was less variability of serum concentrations and also less adverse reactions were seen, including renal injury. A small, retrospective study in critically ill patients found that CI was associated with a faster decrease in leukocyte count and clinical severity score but not with differences in morbidity or mortality [73]. One matched cohort study reported lower mortality rates for critically ill patients with VAP receiving vancomycin in CI (25% vs. 55%). Multiple regression analysis of the data confirmed that CI was associated with improved survival. Unfortunately, this study was not designed to compare CI and II and thus PK/PD data are lacking [74].

Two major clinical studies compared nephrotoxicity between different vancomycin dosing regimens [75,76]. In a study in which 167 outpatients received vancomycin through CI (n = 112) or through II (n = 55), Ingram et al. found that CI is associated with a slower onset, but not a lower prevalence (15.6%), of nephrotoxicity [75]. A nonsignificant tendency toward less nephrotoxicity in favor of CI was seen in 149 patients after cardiac surgery [76].

Byl et al. compared the pleural and serum vancomycin concentrations in 16 patients receiving continuous or intermittent vancomycin infusion and found no difference for AUC_24h_ between the two groups, but antibiotic levels were more sustained with CI [77].

### CI of vancomycin: Discussion

The actual data show no difference in clinical outcome between continuous and intermittent dosing regimens. But for vancomycin, in contrast to the beta-lactams, we expected this; as we have seen, AUC_24h_/MIC is the PK/PD parameter best predictive of clinical efficacy and is dependent only on TDD and renal function.

If not for attaining better clinical efficacy, what other reasons are there for choosing a CI regimen? The study from Wysocki et al. suggests that CI is cheaper, logistically more convenient, achieves target concentrations faster, and results in less variability of the AUC24 compared with II [71]. On a busy nursing ward, it may be easier to obtain a reliable Css than a reliable through concentration, because the first can be obtained at almost any time during the administration period.

The subject of nephrotoxicity is of growing importance, because there is a tendency toward higher, and potentially more nephrotoxic, dosing of vancomycin to attain the advocated PK/PD-target of AUC_24h_ > 400/MIC. As mentioned, two studies have suggested that CI might be associated with a slower onset of nephrotoxicity [75,76]. Wysocki et al. [71] also states that continuous infusion with a target Css of 20–25 mg/L is safe, and a recent meta-analysis of available data found that CI is associated with a significantly lower risk of nephrotoxicity compared with II [78]. However, a recent, retrospective, cohort study of 129 patients who received vancomycin in CI with a target Css of 15–25 μg/mL contradicts the belief that CI is less nephrotoxic than II and finds a high proportion of patients with acute kidney injury (29.5%) [79]. This study had no comparator group of patients with II. Data on toxicity for continuous versus intermittent infusion of vancomycin are conflicting, and no conclusive recommendations can be made [80].

Because there is no evidence for outcome benefit and because data on renal toxicity are conflicting, recent guidelines from the Infectious Diseases Society of America (IDSA) concerning the treatment of methicillin-resistant *Staphylococcus aureus* (MRSA) do not support the generalized use of a continuous infusion for vancomycin in these circumstances [81].

### CI of vancomycin: Practical considerations

Recent consensus recommendations suggest a vancomycin starting dose of 15–20 mg/kg every 8–12 h for II with a loading dose of 25–30 mg/kg [70]. These recommendations for loading dose and TDD can be applied to CI or the dosages can be individualized according to PK/PD targets. The formulas proposed for calculation of individualized dosages are the same as those mentioned for beta-lactam antibiotics. These formulas also suffer from the same shortcomings, as mentioned, for beta-lactam antibiotics and are not readily applicable in critically ill patients. However, they can be used to demonstrate the thin line between therapeutic and toxic vancomycin dosages. For example, attaining an AUC_24h_/MIC of 400 for a patient with a CrCl of 100 ml/min and a pathogen with a MIC of 1 mg/L would require a total daily dose of 2,300 mg of vancomycin. For a MIC of 2 mg/L (the EUCAST susceptibility breakpoint for vancomycin against MRSA), this would mean a total daily dose of 4,500 mg, which approximates a Css of 31.25 mg/L. Ingram et al. [82] showed that the risk for development of acute kidney injury greatly increases when Css exceeds 28 mg/L and 31.5 mg/L is already well above this safety limit. A target Css less than 28 mg/L is achievable only for a pathogen with a MIC of maximal 1.8 mg/L. If the MIC of the pathogen is not known, an empirical target Css of 20–25 mg/L can be used. Jeurissen showed that to achieve a target of 25 mg/L a TDD of 3,000 mg with a loading dose of 1,000 mg should be used in critically ill patients [83]. Because of the difficulties in predicting correctly both the V_d_ and the clearance of vancomycin, it remains advisable to measure serum levels during the first days of therapy so that adjustments can be made rapidly.

Vancomycin is stable at room temperature for at least 24 h and can be given in CI. However, the drug is incompatible with several other drugs, including most notably β-lactams [64]. Clinicians, therefore, are advised to infuse vancomycin through a separate line than the one used for other drugs. Incompatibilities are listed in Table 5. Because of the risk of red-man syndrome, it also is advisable to administer the loading dose in no less than 1–2 h and to use only diluted solutions of vancomycin (typically no more than 10 g/L in 5% glucose).

### Drawbacks of continuous infusion

Most notable disadvantages of continuous infusion are related to the stability of the administered drug, especially for carbapenems, and incompatibilities with other drugs as mentioned before. For carbapenems, this limits their use as “prolonged infusion” (3–4 h), unless solutions are regularly replaced or special precautions are used, such as maintaining the solutions at 4°C. For ceftazidime, the instability issues will become important if the surrounding temperature exceeds 25°C and may necessitate a frequent replacement (every 8 h) of the solutions. In the ICU, the incompatibility problem is partly overcome through the frequent use of multiple lumen central venous catheters. However, on a classic nursing ward where most patients have standard peripheral venous catheters, this can pose practical problems. Clinicians should be advised to check for potential incompatibilities before starting CI (e.g., refer to the lists in [61,62]) and, in case of difficulty, to resort to extended dosing regimens (thus alternating the infusion of the antibiotic with that of the incompatible drug(s).

Another caveat concerns specifically the beta-lactams, which is the risk of neurological toxicity (encephalopathy, convulsions). Beta-lactams are known to cause such adverse effects, but most published studies abut CI did not report its occurrence in patients. Nevertheless, there are anecdotal reports for neurological adverse effects caused by cefepime given by CI (especially if its concentrations exceed 80 mg/L), for reasons that could be related both to the drug intrinsic toxic potential and the liberation of degradation products (see discussion in [62]). Conversely, no adverse effect was noted for temocillin, including in patients with stable levels between 80 and 140 mg/L. Nevertheless, the clinician should remain aware of this risk, especially if deciding to aim at a high serum levels, because of the presence of an organism with a high MIC.

## Conclusions

The pursuit for clinical advantages of CI is still open. There is compelling evidence from PK/PD studies that CI of beta-lactam antibiotics is superior to II for attainment of PK/PD targets. Clinical studies have been less convincing and, although they show at least comparable outcomes between different regimens, they have not been able until now to show a significant benefit in favor of CI. However, several studies show important shortcomings, such as the use of lower antimicrobial dosages in the CI arm. We conclude that CI of beta-lactam antibiotics is not necessarily more advantageous for all patients. The benefit is probably most pronounced in infections with more resistant pathogens and in subpopulations, such as critically ill or immunocompromised patients. This remains to be proven in well-designed, clinical studies with simultaneous in depth PK/PD analysis.

For vancomycin, CI can be chosen, not always for better clinical efficacy, but because it is practical, cheaper, associated with less AUC24h-variability and easier to monitor. Moreover, it might be associated with a slower onset of nephrotoxicity. There is, however, still more to debate about this specific point, and we would strongly encourage more detailed studies in this context.

## Abbreviations

CI: Continuous infusion; II: Intermittent infusion; T > MIC: Time above the minimal inhibitory concentration; PK/PD: pharmacokinetic/pharmacodynamic; MIC: minimal inhibitory concentration; AUC24h: Area under the curve over 24 h; Vd: Volume of distribution; TBC: Total body clearance; RCT: Randomized controlled trial; MSSA: Methicillin susceptible Staphylococcus aureus; VAP: Ventilator-associated pneumonia; EUCAST: European Committee on Antimicrobial Susceptibility Testing; CLSI: Clinical and Laboratory Standards Institute; ICU: Intensive care unit; GFR: Glomerular filtration rate; CrCl: Creatinine clearance; TDD: Total daily dosage; Css: Steady state concentration; LD: Loading dose; Cpeak: Peak concentration; FUO: Fever of unknown origin; NS: Nonsignificant; ITT: Intention to treat; APACHE-II: Acute Physiology and Chronic Health Evaluation.

## Competing interests

The authors declare that they have no competing interests.
